# Clinical significance of postpancreatectomy acute pancreatitis defined by the International Study Group for Pancreatic Surgery

**DOI:** 10.1002/ags3.12587

**Published:** 2022-06-01

**Authors:** Naoki Ikenaga, Kohei Nakata, Nobuhiro Fujita, Toshiya Abe, Noboru Ideno, Kousei Ishigami, Masafumi Nakamura

**Affiliations:** ^1^ Departments of Surgery and Oncology, Graduate School of Medical Sciences Kyushu University Fukuoka Japan; ^2^ Department of Clinical Radiology, Graduate School of Medical Sciences Kyushu University Fukuoka Japan

**Keywords:** pancreas, pancreaticoduodenectomy, pancreatitis, postoperative hyperamylasemia, postpancreatectomy acute pancreatitis

## Abstract

**Aim:**

The International Study Group for Pancreatic Surgery (ISGPS) developed a structured definition of postpancreatectomy acute pancreatitis (PPAP) in 2021. This study aimed to evaluate the clinical significance of PPAP as defined by the ISGPS criteria.

**Methods:**

We evaluated the medical records and postoperative computed tomography (CT) findings of 247 patients who underwent pancreaticoduodenectomy. Postoperative hyperamylasemia (POH) was defined as an elevation in serum amylase levels over the upper baseline limit (≥133 U/L) on postoperative days 1 and 3. PPAP was defined as acute pancreatitis satisfying the following three requirements: POH, clinically relevant deterioration, and radiologic features consistent with acute pancreatitis.

**Results:**

Postoperative hyperamylasemia and PPAP were prevalent in 9.7% (24/247) and 3.6% (9/247) of the patients, respectively. PPAP grade B occurred in eight patients, seven of whom experienced Clavien–Dindo grade IIIA complications, including postoperative pancreatic fistula (POPF) and extended periods of postoperative hospitalization. PPAP grade C occurred in one patient, who died from the exacerbation of underlying interstitial pneumonia following the POPF occurrence. Acute pancreatitis determined by CT was observed in 15.3% (38/247) of the patients who underwent pancreaticoduodenectomy and was strongly associated with severe morbidity (*P* < .0001) and longer postoperative hospitalization (*P* < .0001). POH preceded acute pancreatitis on CT in only 23.7% (9/38) of those cases, resulting in a low incidence rate of PPAP.

**Conclusion:**

Post‐pancreatectomy acute pancreatitis is a major postoperative complication of pancreatic resection; however, based on the current ISGPS criteria, its prevalence is low. Defining PPAP promotes universal evaluation and understanding of this new concept.

## Graphical abstract

The International Study Group for Pancreatic Surgery developed a structured definition of post‐pancreatectomy acute pancreatitis (PPAP) in 2021. We demonstrated the prevalence and clinical significance of PPAP by evaluating medical records and postoperative CT findings of 247 patients who underwent pancreaticoduodenectomy.

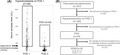

## 1 INTRODUCTION

Emerging evidence has revealed that acute pancreatitis occurs after pancreatic resection due to several causes, including direct operative trauma, transient hypoperfusion, and stasis of pancreatic juice.^1^, ^2^ Several studies have demonstrated that acute pancreatitis, which occurs after pancreatic resection, is associated with a poor postoperative clinical course, especially after pancreaticoduodenectomy (PD).^3^, ^4^, ^5^, ^6^ In a previous study, we reported that postoperative acute pancreatitis, defined by hyperamylasemia on postoperative day (POD) 1, occurred in 63.4% of patients who underwent PD and that patients with hyperamylasemia on POD 1 experienced a significantly increased rate of morbidity compared to those without hyperamylasemia, including postoperative pancreatic fistula (POPF) and prolonged postoperative hospitalization.^4^

Currently, researchers have used different criteria, including biochemical, radiological, and pathological modalities, to evaluate postoperative acute pancreatitis.^2^, ^3^, ^4^, ^5^, ^7^, ^8^, ^9^, ^10^ Hyperamylasemia is one of the easiest to assess and most relevant tools for defining acute pancreatitis. However, the threshold at which serum amylase levels indicate hyperamylasemia and the timing of serum amylase measurement are under debate. Hyperamylasemia defined by the upper limit of normal serum amylase values on POD 1 (Conner's criteria),^4^, ^11^, ^12^ a level three times the upper limit of normal serum amylase values on POD 0‐1 (revised Atlanta classification),^7^, ^13^, ^14^ and sustained serum amylase elevation until POD 2 (dynamic hyperamylasemia)^8^ have been reported to be associated with postoperative morbidity. However, some researchers consider hyperamylasemia to be an insufficient factor for diagnosing postoperative acute pancreatitis because hyperamylasemia is a frequent finding after PD, and most patients with hyperamylasemia did not experience morbidity.^5^, ^15^ These researchers have stated that typical computed tomography (CT) findings indicative of acute pancreatitis are necessary to identify truly dangerous acute pancreatitis after PD.^5^, ^15^

In response to this, the International Study Group for Pancreatic Surgery (ISGPS) proposed diagnostic criteria and a grading system for acute pancreatitis following pancreatic surgery, which they have named postpancreatectomy acute pancreatitis (PPAP).^15^ The diagnosis of PPAP (grade B) requires the following three components: (a) sustained (persisting for at least 48 h) serum amylase activity exceeding the upper limit of baseline values; (b) radiologic features of acute pancreatitis; and (c) clinically relevant changes in management. Persistent organ failure shifts the grade of PPAP from B to C. Unlike conventional postoperative acute pancreatitis, which is diagnosed solely by biochemical criteria, elevations in serum amylase are only a prerequisite to diagnose PPAP, and a sustained increase in serum amylase levels for at least 48 h is considered to indicate postoperative hyperamylasemia (POH). This definition proposed by the ISGPS is novel; therefore, the clinical significance of POH and PPAP has not been assessed to date.

This study aimed to evaluate the clinical significance of the ISGPS definition of PPAP in patients undergoing PD. We investigated the trend of serum amylase levels, the clinical course, and CT findings in a previous cohort^4^ as well as that of additional cases to reveal the prevalence and clinical course of POH and PPAP following the aforementioned surgery.

## 2 METHODS

### 2.1 Patient population

The medical records of 247 consecutive patients who underwent PD (2015–2019) at the Department of Surgery and Oncology, Kyushu University Hospital were retrospectively analyzed. Patients who underwent laparoscopic PD were excluded because this procedure is not well established. The previously collected data from the initial cohort (2015–2017)^4^ included age, sex, body mass index (BMI), American Society for Anesthesiologists physical status, presence of diabetes, neoadjuvant chemotherapy, surgical procedures, combined vascular resection, operation time, blood loss volume, blood transfusions received, texture of the pancreas, pathological diagnosis, serum amylase level on POD 1, serum C‐reactive protein levels on POD 1 and 3, postoperative complications, and length of postoperative hospitalization period, whereas the newly collected data included the use of antibiotics and nutritional support, serum amylase levels on POD 3, and findings of abdominal CT. The data of patients who underwent PD between 2018–2019 were combined with those of the previous cohort for the present analysis. This study was approved by the Institutional Review Board of Kyushu University Hospital (approval number: 2019–089), which waived the requirement for informed consent due to the retrospective nature of this study.

### 2.2 Definitions of clinical parameters

Hyperamylasemia was defined as an elevation in serum amylase levels above the upper limit of baseline values (≥133 U/L). POH, which requires a sustained serum amylase level above the upper limit of baseline values for at least 48 h to be diagnosed according to the PPAP criteria,^15^ was defined as an elevation in serum amylase levels ≥133 U/L on both POD 1 and POD 3 because blood tests were routinely performed in the morning on POD 3, corresponding to a timepoint ~62 h after PD. The level of amylase in drained fluid was routinely measured on POD 1 and 3, and was considered to be zero U/L when the drain was already removed before POD 3. PPAP,^15^ POPF,^16^ biochemical leakage, delayed gastric emptying (DGE),^17^ and postpancreatectomy hemorrhage (PPH)^18^ were defined according to the definitions of the ISGPS. An abdominal abscess was defined as a fluid collection in the abdominal cavity with clinical signs of infection. The severity of complications was determined using the Clavien–Dindo classification system.^19^

All CT findings collected within 30 days after PD in each patient were evaluated retrospectively by a radiologist (N.F.) and a surgeon (N.I.) specializing in the pancreatobiliary region. The diagnosis of acute pancreatitis on CT was determined based on the following typical findings: inflammatory enlargement of the remnant pancreas, interstitial parenchymal edema, inflammatory changes in peripancreatic fat, intra/peripancreatic fluid collections, and parenchymal/peripancreatic necrosis. Acute pancreatitis severity was evaluated according to the modified CT severity index.^20^ This index comprises the following three parameters: (a) pancreatic inflammation (normal, zero points; intrinsic pancreatic abnormalities with or without inflammatory changes in peripancreatic fat, 2 points; and pancreatic or peripancreatic fluid collection or peripancreatic fat necrosis, 4 points); (b) pancreatic necrosis (none, zero points; ≤30%, 2 points; and > 30%, 4 points); and (c) extrapancreatic complications (presence of pleural effusion, ascites, vascular complications, or gastrointestinal tract involvement, 2 points). Pancreatitis severity was categorized as mild (0–2 points), moderate (4–6 points), or severe (8–10 points).

### 2.3 Surgical procedures and postoperative management

Pancreaticoduodenectomy included pylorus‐preserving PD and subtotal stomach‐preserving PD, and reconstruction was performed using the modified Child method. The primary method for performing pancreatojejunostomy was the modified Kakita's method^21^ until the end of March 2015, whereas the modified Blumgart's technique^22^ has been utilized since April 2015. An internal or external pancreatic duct stent was routinely placed in almost all cases, and an external stent was selected for individuals who had a high risk of pancreatic juice leakage. Oral fluid and food intake were initiated on POD 2 and POD 5–7, respectively. Drainage tubes placed during surgery were removed if there were no signs of leakage or bacterial contamination. In principle, neither prophylactic octreotide nor a protease inhibitor was used during the postoperative course. Abdominal contrast‐enhanced CT was performed postoperatively if: (a) there were suspicions of abdominal complications as patients were clinically worsening; or (b) screening for the transparency of the portal vein when portal vein reconstruction was performed.

### 2.4 Statistical analysis

Fisher's exact test was used to evaluate differences in categorical data. Continuous data are presented as median values (interquartile range, IQR) and were analyzed by a Wilcoxon test or logistic regression analysis, when appropriate. A multivariate analysis was performed using the logistic regression model. For the multivariate analysis, the Youden index was calculated to determine the optimal cutoff points of some continuous data variables. A *P*‐value <.05 was considered statistically significant. All statistical analyses were performed with JMP Pro software v. 14.2.0 (SAS Institute, Cary, NC, USA).

## 3 RESULTS

### 3.1 Prevalence and clinical course of POH

**FIGURE 1:**
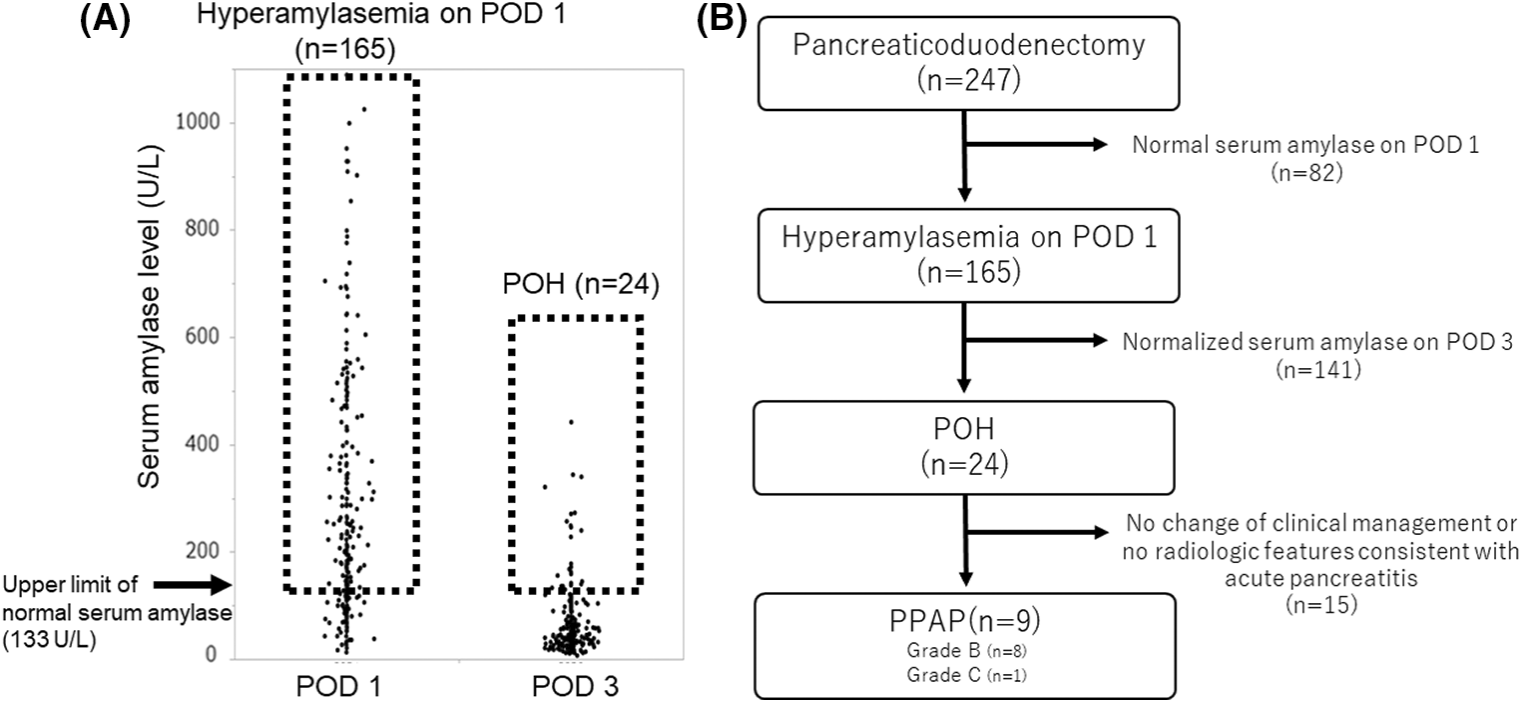
Prevalence of POH and PPAP after PD. (A) Distribution of patients who developed hyperamylasemia on POD 1 and POD 3 after PD. Patients who demonstrated a sustained increase of serum amylase levels above the upper limit of baseline values for more than 48 h (corresponding to POH) comprise 9.7% of the patients who underwent PD. (B) A flow diagram for the diagnosis of PPAP. The incidence rate of PPAP was 3.6% (9/247) among patients who underwent PD. PD, pancreaticoduodenectomy; POD, postoperative day; POH, postoperative hyperamylasemia; PPAP, postpancreatectomy acute pancreatitis

The study population comprised 151 men and 96 women, with a median age of 67 (IQR, 59–73) years. One hundred sixty‐five patients (66.8%) developed hyperamylasemia on POD 1. Of these patients, 24 demonstrated a sustained increase in serum amylase levels over the upper baseline value on POD 3 (Figure 1). Accordingly, the incidence rate of POH after PD was 9.7% (24/247). POH occurrence was significantly correlated with high levels of amylase in the drain fluid on POD 1 and POD 3 (Table 1). Patients who developed POH after PD experienced a significantly increased rate of morbidity, defined as a Clavien–Dindo grade ≥ IIIA (*P* = .0142), including POPF (*P* = .0079). The duration of postoperative hospitalization was not different between the patients with POH and those without (Table 1).

**TABLE 1:** Postoperative outcomes after PD stratified by the occurrence of POH

	Non‐POH (n = 223)	POH (n = 24)	*P* value
Clavien–Dindo (≥IIIA)	41 (18.4%)	10 (41.7%)	.0142*
POPF (≥grade B)	32 (14.4%)	9 (37.5%)	.0079*
BL or POPF	86 (38.6%)	20 (83.3%)	<.0001*
DGE (≥grade B)	33 (14.8%)	2 (8.3%)	.5447
PPH (≥grade B)	2 (0.9%)	1 (4.2%)	.2651
Abscess	30 (13.5%)	8 (33.3%)	.0172*
In‐hospital mortality	0	1 (9.4%)	.0972
Postoperative hospitalization (days)	19 (16–33)	22 (17–35)	.1164
C‐reactive protein (mg/dL)			
POD 1	10.5 (8.3–13.8)	9.5 (8.7–13.4)	.8663
POD 3	11.8 (7.4–18.3)	15.4 (9.2–23.7)	.0475*
Amylase in drain fluid (U/L)			
POD 1	429 (73–2586)	2056 (1199–5878)	.0006*
POD 3	161 (0–1153)	1613 (469–4104)	.0013*

Abbreviations: BL, biochemical leakage; DGE, delayed gastric emptying; PD, pancreaticoduodenectomy; POD, postoperative day; POH, postoperative hyperamylasemia; POPF, postoperative pancreatic fistula; PPH, postpancreatectomy hemorrhage.

*P values are statistically significant.

### 3.2 Prevalence and clinical course of PPAP

**FIGURE 2:**
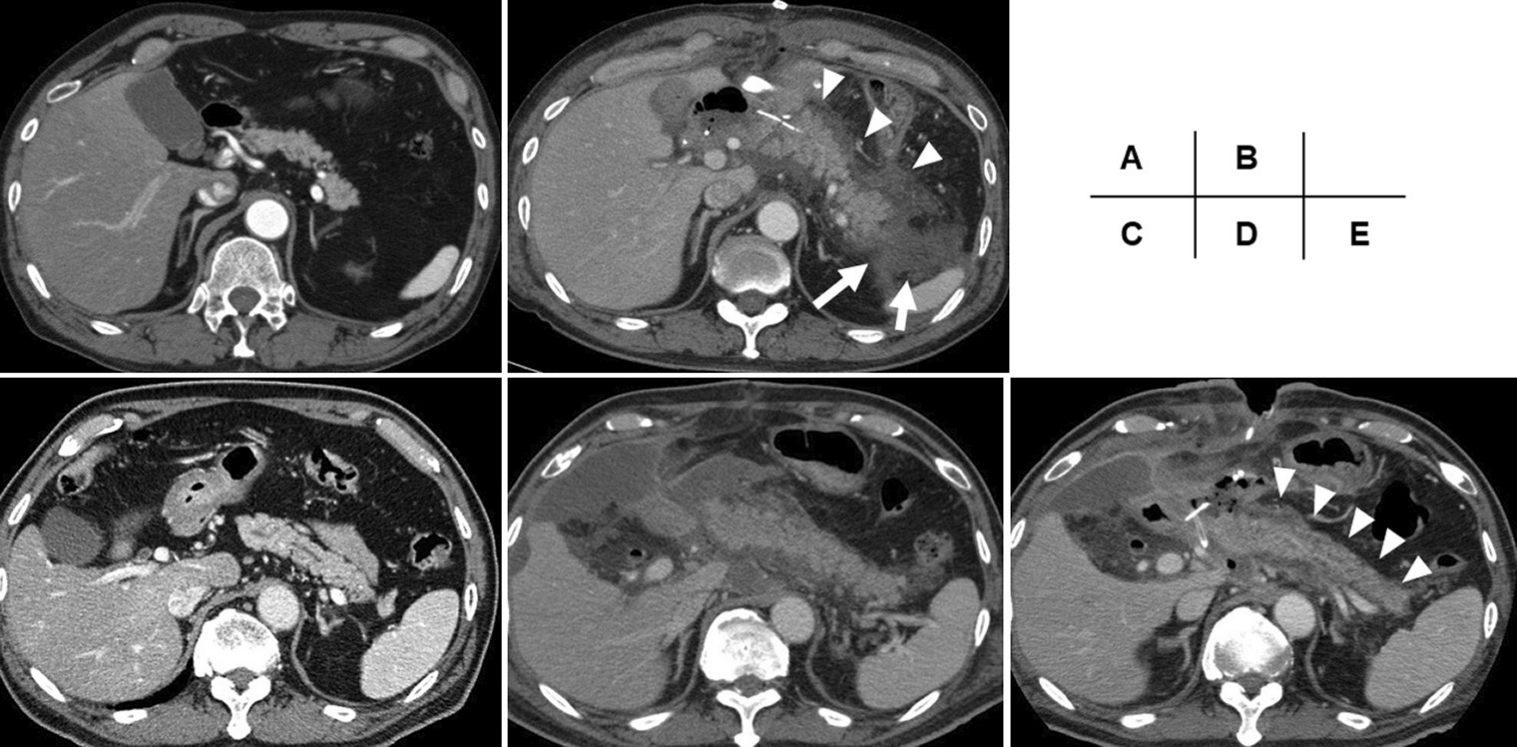
Preoperative (A,C) and postoperative (B,D,E) CT findings in patients who developed PPAP. Preoperative (A) and postoperative (B) CT findings in a patient who developed moderate acute pancreatitis after PD. (B) The remnant pancreas taken on POD 7 demonstrating pancreatic enlargement, broad inflammatory changes in peripancreatic fat (arrowheads), and peripancreatic fluid collection (arrows). Pancreatic necrosis has not occurred. Preoperative (C) and postoperative (D,E) CT findings in a patient who developed severe acute pancreatitis after PD. (D) The remnant pancreas in the CT taken on POD 12 demonstrating diffuse inflammatory pancreatic enlargement, inflammatory changes in peripancreatic fat, peripancreatic fluid collection, and ascites. (E) The enhancement of the remnant pancreas has weakened on POD 19 (arrowheads), indicating that the parenchyma of the pancreas turned to necrosis. CT, computed tomography; PD, pancreaticoduodenectomy; POD, postoperative day; PPAP, postpancreatectomy acute pancreatitis

Among the 24 patients who developed POH, 12 underwent abdominal contrast‐enhanced CT because of clinical deterioration, including sudden fever, abdominal pain, and purulent discharge. Nine patients demonstrated a finding consistent with acute pancreatitis, including mild pancreatitis in four patients, moderate pancreatitis in two patients, and severe pancreatitis in three patients (Figure 2). All patients who developed acute pancreatitis required antibiotics or nutritional support; accordingly, the incidence rate of PPAP was 3.6% (9/247) among patients who underwent PD. Eight patients who developed PPAP did not develop persistent organ failure, require reoperation, or experience death, which led us to confirm that the severity of PPAP in those individuals was grade B. Seven of the eight patients who developed PPAP grade B experienced Clavien–Dindo grade IIIA complications, including POPFs and abdominal abscesses, resulting in extended periods of postoperative hospitalization in patients with PPAP grade B (median, 35 days; IQR, 30–44 days). One patient who developed PPAP died due to the exacerbation of underlying interstitial pneumonia following POPF occurrence 169 days after undergoing PD. Consequently, the incidence rate of PPAP grade C was 0.4% (1/247). Figure 1B outlines the prevalence of POH and PPAP with a flow diagram.

### 3.3 Prevalence and clinical course of postoperative acute pancreatitis determined by CT

**FIGURE 3:**
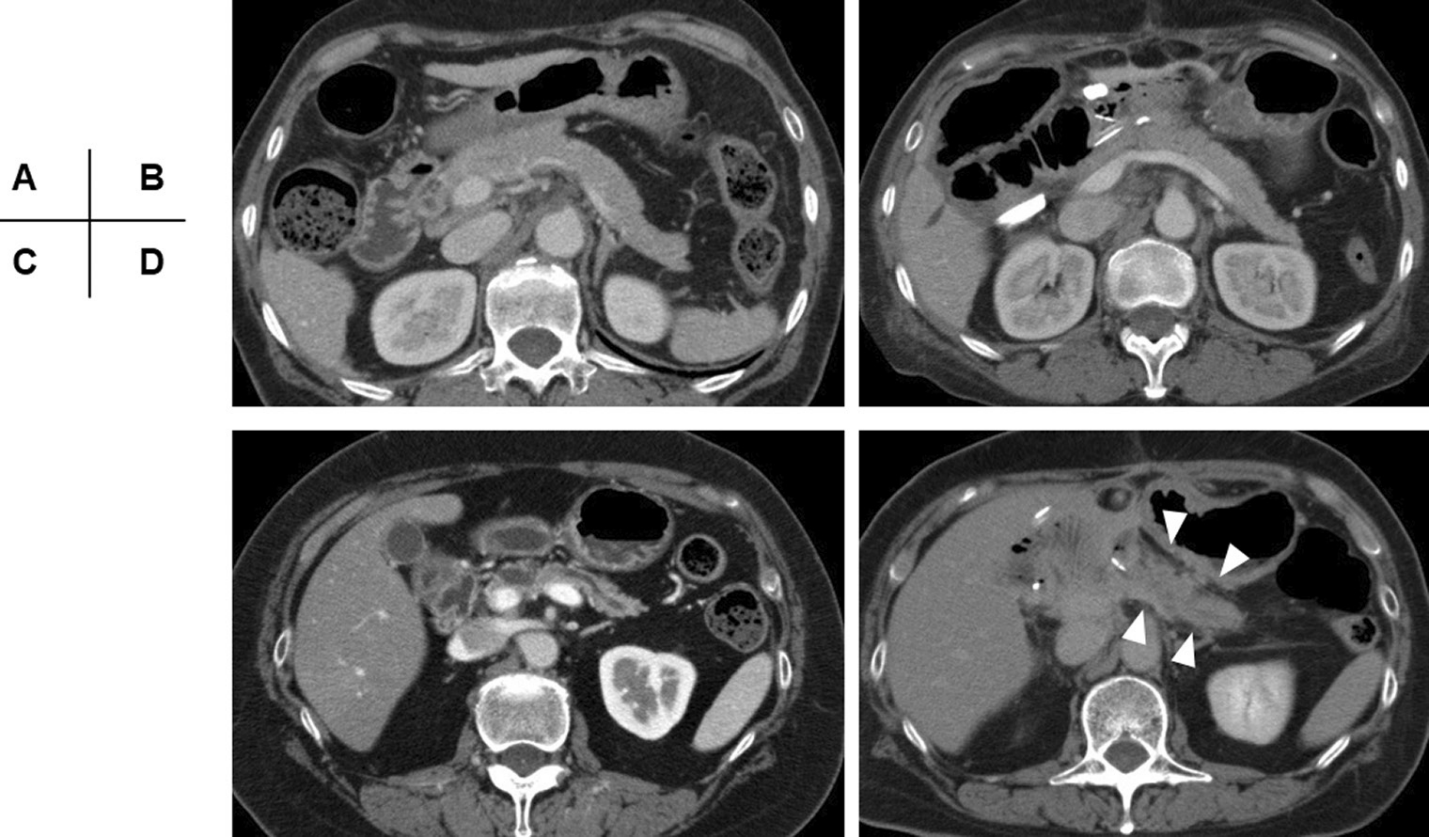
Preoperative (A,C) and postoperative (B, D) CT findings in patients who underwent PD. Preoperative (A) and postoperative (B) CT findings in a patient who did not develop acute pancreatitis after PD. (B) The remnant pancreas on the CT examination taken on POD 8 has not changed compared with the pancreas on preoperative CT. Preoperative (C) and postoperative (D) CT findings in the patients who developed mild acute pancreatitis after PD. the remnant pancreas on the CT taken on POD 24 demonstrates interstitial edema and inflammatory changes in peripancreatic fat (arrowheads). CT, computed tomography; PD, pancreaticoduodenectomy; POD, postoperative day

Among the 247 patients who underwent PD, 90 underwent abdominal contrast‐enhanced CT postoperatively. Thirty‐eight patients (15.3% in patients with PD) demonstrated typical findings of acute pancreatitis on CT examinations, including mild pancreatitis in 15 patients, moderate pancreatitis in 19 patients, and severe pancreatitis in four patients (Figure 3). All patients with acute pancreatitis detected via CT required a change in clinical management, such as administration of antibiotics and/or nutritional support, to treat clinical deterioration. A protease inhibitor was administered to one patient who developed severe acute pancreatitis and not to the other patients. Compared to patients without CT‐determined acute pancreatitis, those with CT‐determined acute pancreatitis experienced a significantly higher rate of POPF (65.8% vs 7.7%, *P* < .0001) and severe morbidity (76.3% vs 10.5%, *P* < .0001), resulting in a significantly longer post‐PD hospitalization duration (42 days vs 18 days, *P* < .0001) (Table 2). The date at which a CT examination revealed acute pancreatitis ranged from POD 4 to POD 24, with a median of POD 10 (Figure S1).

**TABLE 2:** Postoperative outcomes after PD stratified by the occurrence of postoperative acute pancreatitis determined by CT

	CT‐determined acute pancreatitis		
	No (n = 209)	Yes (n = 38)	*P* value
Clavien–Dindo (≥IIIA)	22 (10.5%)	29 (76.3%)	<.0001*
POPF (≥ grade B)	16 (7.7%)	25 (65.8%)	<.0001*
BL or POPF	71 (34.0%)	35 (92.1%)	<.0001*
DGE (≥ grade B)	29 (13.9%)	6 (15.8%)	.8004
PPH (≥ grade B)	1 (0.5%)	2 (5.3%)	.0626
Abscess	19 (9.1%)	19 (5‐00.0%)	<.0001*
In‐hospital mortality	0	1 (2.6%)	.1538
Postoperative hospitalization (days)	18 (16–24)	42 (32–49)	<.0001*

Abbreviations: BL, biochemical leakage; DGE, delayed gastric emptying; PD, pancreaticoduodenectomy; POD, postoperative day; POPF, postoperative pancreatic fistula; PPH, postpancreatectomy hemorrhage.

*P values are statistically significant.

**FIGURE 4:**
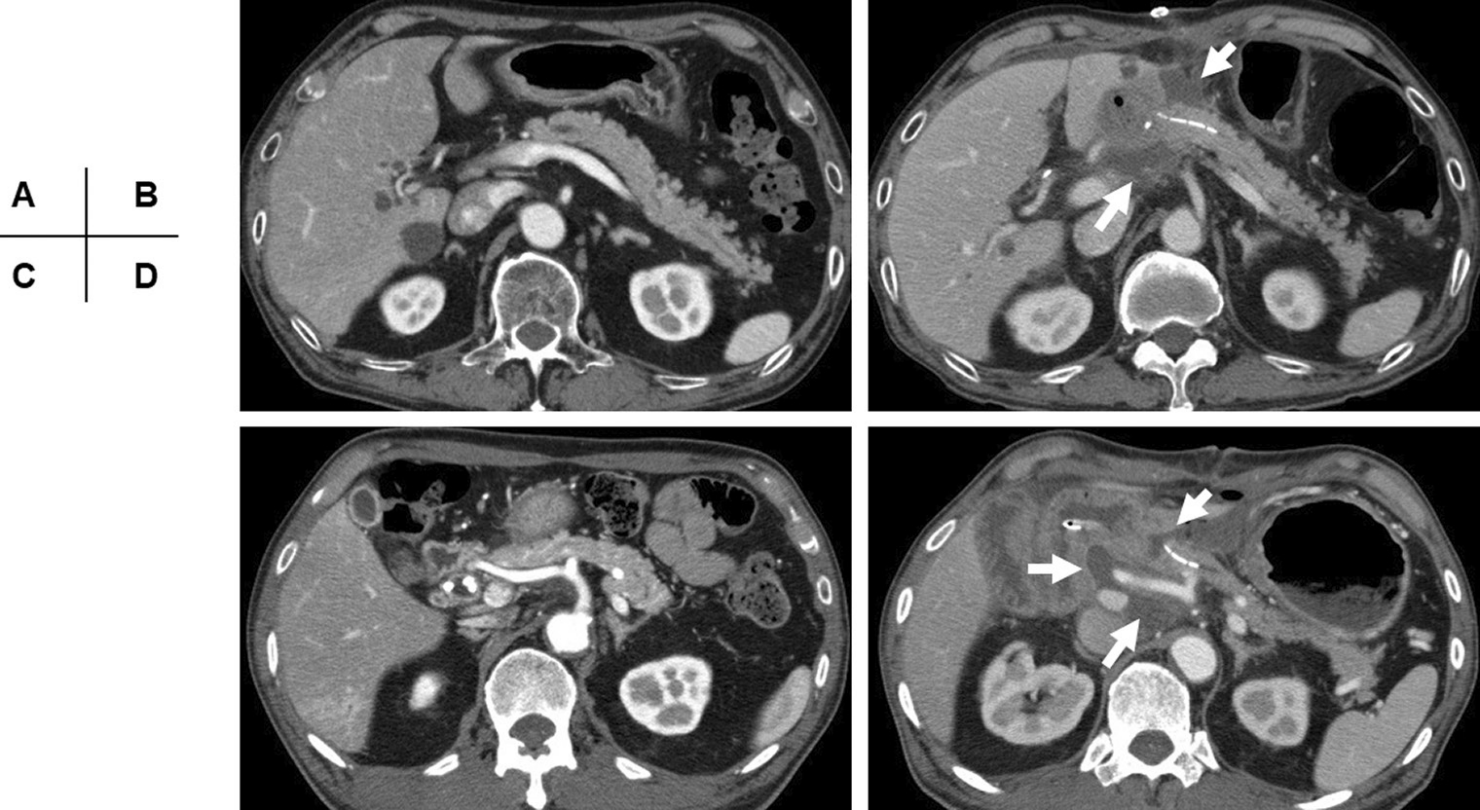
Representative CT findings of POPF without postoperative acute pancreatitis. Preoperative (A,C) and postoperative (B,D) CT findings in each patient. (B,D) postoperative CT evaluations demonstrating fluid collection solely around the pancreatojejunostomy (arrows) that is not accompanied by morphological changes in the remnant pancreas. Peripancreatic inflammatory changes are also not observed. CT, computed tomography; POPF, postoperative pancreatic fistula

In 19 of 90 patients who underwent CT after PD, fluid collection was observed only around the pancreatojejunostomy, while the morphology of the remnant pancreas and peripancreatic fat were comparable to their preoperative state (Figure 4). This fluid collection solely in that location was considered a leakage of pancreatic juice but not a typical finding of acute pancreatitis. Thirteen of these 19 patients eventually developed POPF.

### 3.4 Clinical significance of hyperamylasemia on POD 1

**FIGURE 5:**
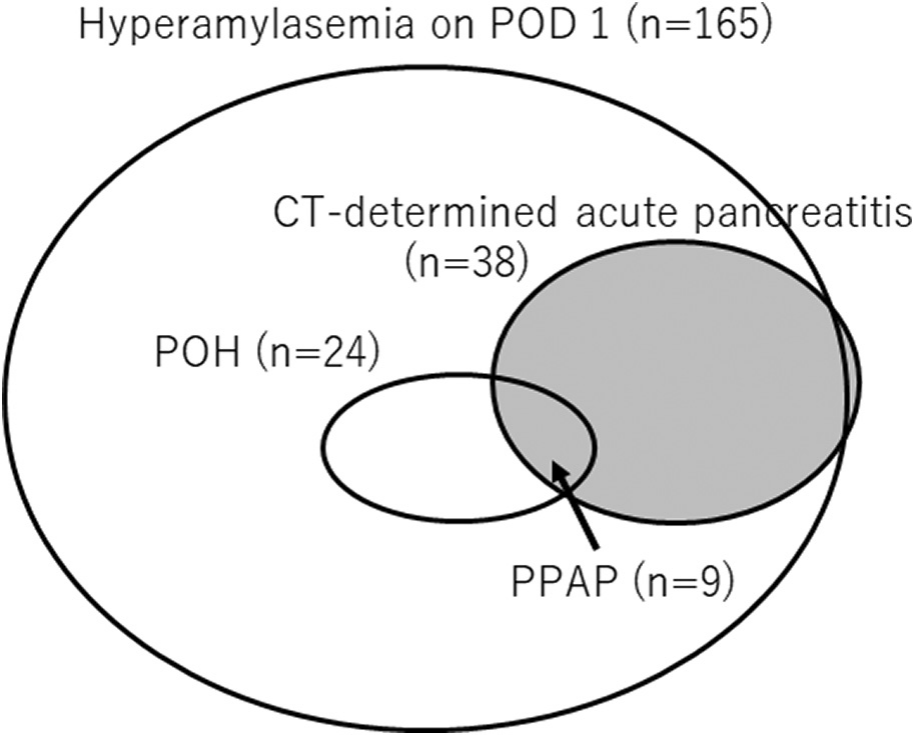
Euler diagram illustrating the correlation between POH, PPAP, CT‐determined acute pancreatitis, and hyperamylasemia on POD 1. The population demonstrating POH and hyperamylasemia on POD 1 is shown by outlined circles. The population demonstrating CT‐determined acute pancreatitis is illustrated by a gray circle. The overlap area of POH and acute pancreatitis on CT indicates PPAP. CT, computed tomography; POD, postoperative day; POH, postoperative hyperamylasemia; PPAP, postpancreatectomy acute pancreatitis

Among the 38 patients with acute pancreatitis determined by CT, 36 (94.7%) experienced preceding hyperamylasemia on POD 1. However, only nine (23.7%) patients demonstrated preceding POH because the serum amylase level had normalized on POD 3 in 27 patients that exhibited acute pancreatitis on CT (Figure 5). The multivariate analysis revealed that a high BMI, hyperamylasemia on POD 1, and POH were independent predictors of acute pancreatitis (Table 3). Among these factors, hyperamylasemia on POD 1 showed the highest odds ratio of 6.8 as a predictor of acute pancreatitis. One hundred forty‐one (85.5%) of 165 patients who developed hyperamylasemia on POD 1 did not meet the criteria for POH, as their serum amylase levels normalized on POD 3 (Figure 1). Patients with hyperamylasemia on POD 1 and normalized serum amylase levels on POD 3 still experienced a significantly higher rate of CT‐determined acute pancreatitis (19.2% vs 2.4%, *P* = .0002) and severe morbidity (24.8% vs 7.3%, *P* = .0011), resulting in a longer postoperative hospitalization period (21 days vs 17 days, *P* < .0001) than in patients without an increase in serum amylase levels postoperatively (Table S1).

**TABLE 3:** Predictors of postoperative acute pancreatitis determined by CT

	Univariate analysis			Multivariate analysis		
	No (n = 209)	Yes (n = 38)	*P* value	OR	CI 95%	*P* value
ASA (1/2/3)	26/172/11	3/27/8	.2109			
Sex (F/M)	86/123	10/28	.1038			
BMI (kg/m^2^)	21.1 (19.6–23.4)	22.7 (21.2–25.1)	.0051*	2.3	1.07–4.83	.0333*
Diabetic	51 (24.4%)	11 (29.0%)	.5466			
Neoadjuvant chemotherapy	68 (32.5%)	7 (18.4%)	.0878			
Procedure (PD/PPPD/SSPPD)	16/102/91	5/16/17	.4825			
Vascular resection	38 (18.2%)	5 (13.2%)	.6417			
Pancreatojejunostomy (Kakita/Blumgart/others)	36/150/23	6/27/5	.9201			
Pancreatic duct stent (internal/external/none)	143/56/10	24/13/1	.6477			
Operation time (min)^a^	361 (292–422)	411 (328–489)	.0036*	3.3	0.92–11.7	.0661
Blood loss volume (mL)	600 (392–917)	686 (449–1081)	.1937			
Soft texture	75 (35.9%)	23 (60.5%)	.0064*	1.3	0.58–2.82	.5381
Pathology (PDAC/others)	126/83	17/21	.1070			
Hyperamylasemia on POD 1	129 (61.7%)	36 (94.7%)	<.0001*	6.8	1.48–31.7	.0139*
POH	15 (7.2%)	9 (23.7%)	.0044*	2.8	1.05–7.59	.0390*

Abbreviations: ASA, American Society for Anesthesiologists physical status; BDAC, bile duct adenocarcinoma; BMI, body mass index; CI, confidence interval; OR, odds ratio; PDAC, pancreatic ductal adenocarcinoma; POD, postoperative day; POH, postoperative hyperamylasemia.

*P values are statistically significant.

^a^ Operation time ≥300 min was used as a cutoff point for multivariate analysis.

## 4 DISCUSSION

This study revealed that POH and PPAP occurred with a relatively low frequency after pancreatic resection. POH occurrence was significantly correlated with POPF development, and all patients who developed PPAP experienced severe morbidity. Postoperative acute pancreatitis determined by CT was strongly correlated with a poor clinical course; however, most cases of acute pancreatitis were not preceded by POH. To the best of our knowledge, this study is the first to report the clinical significance of PPAP as newly defined by ISGPS in 2021 compared to that of CT‐confirmed acute pancreatitis.

Hyperamylasemia based on a single postoperative measurement on POD 0 or POD 1 has been used to define postoperative pancreatitis. It is known to predict major complications after PD.^3^, ^4^, ^5^, ^6^, ^11^ Recently, Bannone et al^8^ revealed that a sustained elevation in serum amylase activity up to POD 2 predicts complications better than a peak elevation of serum amylase on POD 0–1. This report suggests that the dynamic measurement of serum amylase activity could be useful for identifying clinically relevant postoperative acute pancreatitis. Generally, serum amylase activities show a dynamic trend of peaking on POD 1, followed by a decrease to normal values on POD 3–5.^5^, ^8^ Therefore, the timing of measurements of serum amylase activity is important to determine whether there is hyperamylasemia. ISGPS has defined POH as hyperamylasemia persisting for at least 48 h postoperatively, with a serum amylase level greater than the institutional upper limit of baseline values. When applying this definition to our cohort, POH was found in only 9.7% of the patients who underwent PD. However, patients who developed hyperamylasemia on POD 1 but demonstrated normalized serum amylase values on POD 3 still experienced more acute pancreatitis and major complications than those who did not have hyperamylasemia. This indicates that focusing solely on patients with POH could cause one to overlook patients experiencing a poor clinical course. Hyperamylasemia on POD 1 as an indicator could apply to more patients developing acute pancreatitis and severe complications than POH. Patients with hyperamylasemia on POD 1 should be observed carefully, even if their amylase levels have normalized on POD 3.

Emerging evidence has indicated that acute inflammatory changes in the remnant pancreas induced by surgical manipulation led to pancreatic necrosis and the development of POPF.^3^, ^9^, ^10^, ^11^, ^23^ We carefully evaluated CT findings after PD in this study. We observed that postoperative acute pancreatitis and POPF can exist separately, even though they are closely related. It is difficult to distinguish between single fluid collection derived from POPF and single fluid collection as a characteristic of acute pancreatitis, because the leakage of pancreatic juice can induce inflammatory changes in surrounding tissues. Note that the key to distinguishing between the aforementioned factors is parenchymal changes in the remnant pancreas, such as interstitial edema, inflammatory enlargement, and, possibly, a decreased enhancement of the pancreas, as these factors represent direct changes caused by parenchymal inflammation or necrosis. Inflammatory changes in peripancreatic fat apart from the anastomotic portion are also useful for diagnosing acute pancreatitis after PD. Our present study described landmarks for interpreting acute pancreatitis after pancreatic resection using abundant images based on the statement regarding the radiologic features of PPAP by the ISGPS.

Our study revealed that most patients with acute pancreatitis determined by CT demonstrated hyperamylasemia on POD 1, whereas only 23.7% of such patients demonstrated POH. Consequently, the population that satisfied the criteria for PPAP comprised only 3.6% of the patients who underwent PD. The current criteria for diagnosing POH provide a narrow focus of attention and could overlook the population requiring care during the postoperative course of PD. Consequently, PPAP has become a rare condition that could lead to an underestimation of clinically relevant acute pancreatitis. The ISGPS states that PPAP grading refers to a retrospective assessment of the severity of the complication and is not a prospective treatment proposal.^15^ In other words, the definition of PPAP proposed by the ISGPS is not intended to predict a poor clinical course after pancreatic resection; rather, it facilitates the standardization of criteria for evaluating this newly identified postpancreatectomy complication. Similar to that of other postpancreatectomy complications such as POPF, DGE, and PPH, a consensus statement regarding PPAP would promote the future understanding of the etiology and treatment strategies for PPAP.

Limitations of this study include the lack of data for serum amylase levels on POD 2. Blood examination in the morning on POD 2 corresponds to a timepoint 34 h after pancreatic resection; therefore, hyperamylasemia on POD 2 does not meet the criteria for POH. However, if we assume that an elevated serum amylase level on POD 2 is POH, the incidence rate of POH and PPAP would become higher compared with the present results. In that case, POH could cover a wider population developing acute pancreatitis. Other limitations of this study are its retrospective nature and that CT examination was not conducted in all patients who underwent PD. Therefore, the real incidence rate of acute pancreatitis after PD could not be elucidated. Occult postoperative acute pancreatitis might have been overlooked in patients who did not undergo CT examination.

## 5 CONCLUSION

Postpancreatectomy acute pancreatitis is one of the major complications that may occur after pancreatic resection; however, its incidence rate is relatively low. The value of the definition of PPAP is that it enables the standardization required for universal evaluation and outcome comparison of PPAP across different studies, increasing the understanding of this novel concept.

## DISCLOSURE

Conflict of Interest: There are no conflicts of interest to declare.

Author Contributions: Study concept and design: N.I. Acquisition of data: N.I., N.F., K.N., T.A., and N.I. Statistical analysis and interpretation of data: N.I. and N.F. Drafting of the article: N.I. and M.N. Critical revision of the article for important intellectual content: all authors. Supervision: K.I. and M.N.

Ethics Statement: This study was approved by the Institutional Review Board of the Kyushu University Hospital (approval no. 2019–089), and it conforms to the provisions of the Declaration of Helsinki. Informed consent was waived owing to the retrospective nature of the study. The opt‐out recruitment method was applied to all patients, with an opportunity to decline to participate.

## Supporting information


Figure S1

Table S1
