# Supplementary figures and images for: Clinical significance of postpancreatectomy acute pancreatitis defined by the International Study Group for Pancreatic Surgery
Source: Ann Gastroenterol Surg. 2022 Jun 1;6(6):842–50. doi: 10.1002/ags3.12587 (PMC9628230; doi:10.1002/ags3.12587)

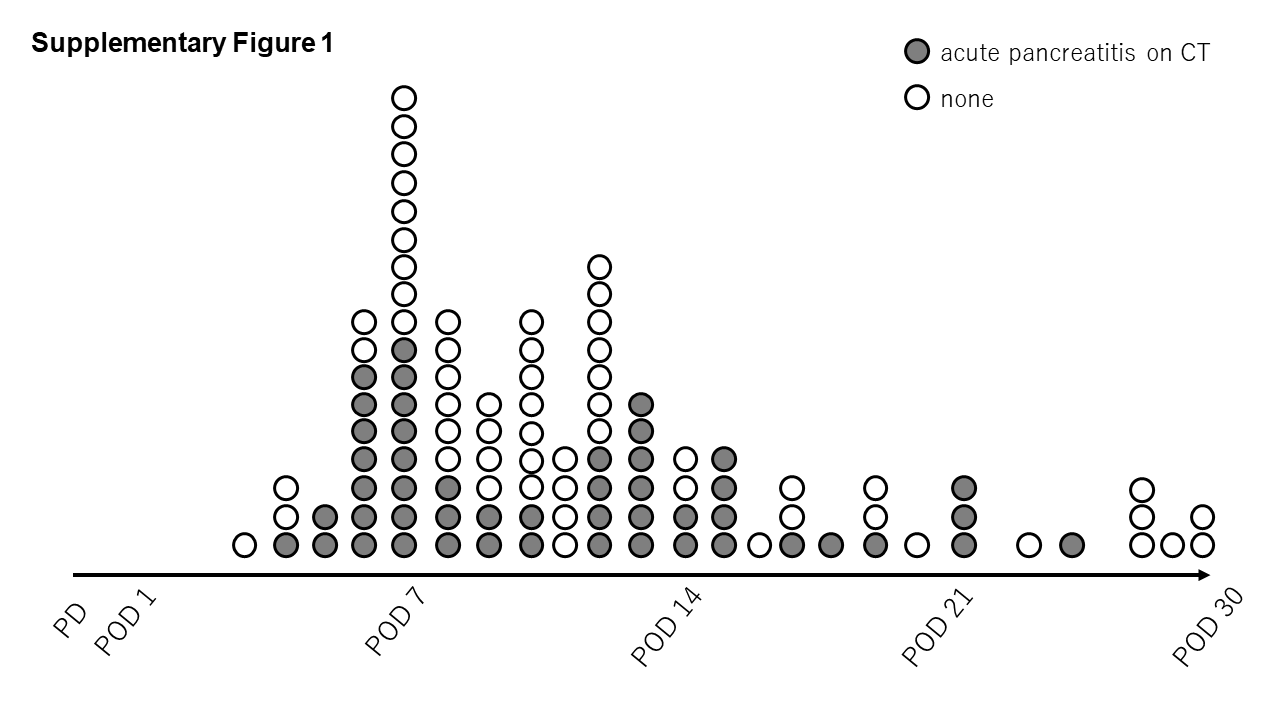

Supplement: Supplementary file 1 — Figure S1 [file AGS3-6-842-s001.TIF]
