# Supplementary material for: Clinical significance of postpancreatectomy acute pancreatitis defined by the International Study Group for Pancreatic Surgery
Source: Ann Gastroenterol Surg. 2022 Jun 1;6(6):842–50. doi: 10.1002/ags3.12587 (PMC9628230; doi:10.1002/ags3.12587)
Supplement: Supplementary file 2 — Table S1 [file AGS3-6-842-s002.docx]

**SUPPORTING INFORMATION**

**Supplementary Table 1. Postoperative outcomes after PD in the patients with hyperamylasemia on POD 1 and normalized amylase level on POD 3.**

|  | Non-POH patients (n=223) | |  |
| --- | --- | --- | --- |
|  | Normal (n=82) | Hyperamylasemia on POD1  (n=141) | P value |
| CT-determined  acute pancreatitis | 2 (2.4%) | 27 (19.2%) | 0.0002* |
| Clavien-Dindo (≥IIIA) | 6 (7.3%) | 35 (24.8%) | 0.0011* |
| POPF (≥ grade B) | 2 (2.4%) | 30 (21.3%) | <.0001* |
| BL or POPF | 5 (6.1%) | 81 (57.5%) | <.0001* |
| DGE (≥ grade B) | 13 (15.9%) | 20 (14.2%) | 0.8452 |
| PPH (≥ grade B) | 1 (1.2%) | 1 (0.7%) | 1.0000 |
| Abscess | 2 (2.4%) | 28 (19.9%) | 0.0002* |
| Mortality on POD 90 | 0 | 0 | N/A |
| Postoperative hospitalization (days) | 17 (15–22) | 21 (16–37) | <.0001* |
| C-reactive protein (mg/dL) |  |  |  |
| POD 1 | 10.4 (8.2–13.2) | 10.5 (8.5–14.1) | 0.5005 |
| POD 3 | 9.4 (6.7–12.6) | 14.2 (8.4–20.6) | <.0001* |
| Amylase in drain fluid (U/L) |  |  |  |
| POD 1 | 73 (41–155) | 1891 (347–3,609) | <.0001* |
| POD 3 | 0 (0–35) | 661 (99–2,692) | <.0001* |

PD, pancreaticoduodenectomy; POH, postoperative hyperamylasemia; POPF, postoperative pancreatic fistula; BL, biochemical leakage; DGE, delayed gastric emptying; PPH, post-pancreatectomy hemorrhage; POD, postoperative day

**Supplementary Figure 1. Time sequence of CT examination after PD**

Circles indicate the date when CT examinations were conducted. Acute pancreatitis was frequently identified between POD 6 and POD 15. Multiple CT examinations conducted on different days in the same patients are shown separately.

CT, computed tomography; PD, pancreaticoduodenectomy; and POD, postoperative day
